# The NRW80+ study: conceptual background and study groups

**DOI:** 10.1007/s00391-021-01970-z

**Published:** 2021-09-27

**Authors:** Sylvia Hansen, Roman Kaspar, Michael Wagner, Christiane Woopen, Susanne Zank

**Affiliations:** 1 Ceres—Cologne Center for Ethics, Rights, Economics, and Social Sciences of Health, University of Cologne, Albertus Magnus Platz, 50923 Cologne, Germany; 2 University of Cologne, Institute of Sociology and Social Psychology, Cologne, Germany; 3 University of Cologne, Faculty of Humanities, Rehabilitative Gerontology, Cologne, Germany

**Keywords:** Representative survey, Very old age, Quality of life, Cohort, Age groups, Repräsentative Umfrage, Hochaltrigkeit, Lebensqualität, Kohorte, Altersgruppen

## Abstract

**Background:**

The study “Quality of life and well-being of the very old in North Rhine-Westphalia NRW80+” aims at giving a representative picture of the quality of life (QoL) in this population. Conceptually, QoL research has rarely considered the values of older individuals themselves and societal values, and their relevance for successful life conduct. Empirically, comparisons of different age groups over the age of 80 years are rare and hampered by quickly decreasing numbers of individuals in oldest age groups in the population of very old individuals.

**Study design and theoretical framework:**

This paper describes the population of the NRW80+ study and different age groups of very old individuals with respect to biographical background. Furthermore, using the challenges and potentials model of QoL in very old age (CHAPO), key aspects of QoL in late life are discussed and the importance of normative stipulations of what constitutes a successful life conduct are highlighted. In the NRW80+ study older age groups (i.e., 85–89 years, 90+ years) were deliberately overrepresented in the survey sample to enable robust cross-group comparison. Individuals willing to participate in the study but unable to participate in the interview themselves for health reasons were included by means of proxy interviews.

The total sample included 1863 individuals and 176 individuals were represented by proxy interviews. Pronounced differences were observed between age groups 80–84 years (born 1933–1937, *N* = 1012), 85–89 years (born 1928–1932, *N* = 573), and 90 years or older (*born before 1927, *N* = 278) with respect to education, employment and the timing of major life events (e.g., childbirth).

**Conclusion:**

Different life courses and resulting living conditions should be considered when discussing QoL disparities in very old age.

## Abstract (de)

**Hintergrund:**

Die Studie „Lebensqualität und Wohlbefinden hochaltriger Menschen in Nordrhein-Westfalen (NRW80+)“ zeichnet ein repräsentatives, differenziertes Bild der Lebensqualität (LQ) in der Hochaltrigkeit. Bisher vorgeschlagene LQ-Modelle berücksichtigen nur unzureichend sowohl individuelle Werthaltungen älterer Menschen als auch gesellschaftliche Werte bei der Diskussion von Konstellationen gelingender Lebensführung. Nur wenige empirische Studien erlauben gegenwärtig einen differenzierten Blick auf Altersunterschiede jenseits von 80 Jahren, nicht zuletzt wegen des geringen Anteils der Ältesten in der Population.

**Studiendesign/Rahmenmodell:**

Der Artikel beschreibt die NRW80+-Studienpopulation und Altersgruppen hinsichtlich ihres biografischen Hintergrunds. Die Nutzung des Challenges and Potentials Model of QoL in Very-Old Age (CHAPO) erlaubt es, Kernpunkte von LQ zu erörtern und die Relevanz von normativen Wertevorstellen über eine erfolgreiche Lebensführung herauszustellen. Ältere Hochaltrige wurden in der Stichprobe gezielt überrepräsentiert, um aussagekräftige Altersvergleiche zu erlauben. Informationen über Personen, die aufgrund ihres Gesundheitszustands das Interview nicht selbst führen konnten, wurden durch Stellvertreterinterviews mit einem nahen Angehörigen/Bekannten erhoben. Die Gesamtstichprobe umfasste 1863 Interviews; für 176 Personen wurden Stellvertreterinterviews geführt. Die betrachteten Kohorten 80–84 Jahre (geb. 1933–1937, *N* = 1012), 85–89 Jahre (geb. 1928–1932, *N* = 573) und 90 Jahre oder älter (geb. vor 1927, *N* = 278) unterschieden sich wesentlich mit Blick auf Lebensverlauf und grundlegende Lebensumstände.

**Schlussfolerung:**

Unterschiedliche Lebensverläufe und resultierende Lebensbedingungen sollten bei der Diskussion von ungleicher LQ im sehr hohen Lebensalter berücksichtigt werden.

The goal of the study “Quality of Life and Well-Being of the Very Old in North Rhine-Westphalia NRW80+” is to provide a representative picture of quality of life (QoL) in the population of those 80 years or older [40]. This paper serves as an introduction to the thematic focus of this special issue, providing a basic understanding of the NRW80+ study and sample. All papers in this issue are based upon NRW80+ data. The aim of this introduction is twofold. First, a brief characterization of the targeted population is offered with respect to biographical background, historical context, and the age structure of today’s very old individuals in NRW. Second, key aspects of the “Challenges and Potentials Model of Quality of Life in Very Old Age (CHAPO)” are discussed and the importance of stipulations of what constitutes the good life or successful life conduct are highlighted.

## The population of very old individuals today

There is no single agreed upon definition of “very old age”. In the NRW80+ study, the definition of very old age as a chronological age of 80 years or older has been chosen primarily for pragmatic reasons, as it is often the case in population-based surveys [17, 27, 40]. It has been shown that from about 80 years onwards, the probability of a variety of age-associated changes such as health impairments increases. This has led to the well-known distinction between resource-rich third age and resource-poor fourth age [1, 2]. Due to achievements in healthcare, social life, and technical advances, some scholars argue that today, people in their 60s or 70s no longer correspond to traditional understandings of old age. Rather, the fourth age appears to be the real age that bears strong resemblance to classical (negative) views on old age. Nevertheless, aging and old age can also be associated with positive aspects such as rich experience, accumulated knowledge, and serenity [25, 33]. For a comparative overview of perspectives on the third and fourth age and risks of such a distinction, see Wahl and Ehni [41].

Today, life beyond 80 years of age may span one or even two more decades for many individuals, making the very old a group that comprises a great number of diverse birth cohorts. It is paramount to understand differences in early socialization, education, and life experiences as potential determinants of QoL outcomes in very old age; however, a comparison of age groups within very old age is hampered by quickly decreasing numbers of very old and oldest individuals in the population and a growing disproportionality of men and women particularly in the oldest age groups. As a consequence, many empirical studies offer limited possibilities to differentiate age groups within very old age, even if they do not specify a maximum age for study participation [5]. In NRW80+, three groups of very old people were considered: 80–84 years (born 1933–1937), 85–89 years (born 1928–1932), and 90 years or older (born before 1927).

Reference studies in the field of aging research (e.g. BASE, SHARE, German Ageing Study) have shown that the group of older people is very heterogeneous with respect to, for example, functional status [22] or social engagement [20]. Such interindividual differences may be due to differences in life courses. It has been shown that earlier life experiences influence not only health but also QoL in later life [3, 19, 30]. People’s life courses are influenced by societal factors such as political decisions and historical circumstances happening at a certain time and experienced at different times in their life course.

For today’s oldest old, one important historical event was the Second World War (WWII) and its consequences. All NRW80+ age groups were socialized during times of National Socialism and war; however, participants aged 80–84 years and 85–89 years today were often young enough to be part of the Nazi evacuation scheme and may have participated as soldiers only towards the end of WWII. Older age groups were likely to have been more actively involved in war-related combat or consequences of the war in the home country. The post-war period was characterized by overcoming the traumas from the war period. The younger age groups may have been more influenced by the economic upswing and the worldviews of the Allied Forces.

In general, the older age groups (85+ years) attained fewer years of education due to the war. A large percentage of this age group left school early, attaining lower secondary education at best, whereas the individuals of the younger age group usually reached higher educational qualifications [24]. Consequently, men born around 1930 had difficulties finding apprentice positions or take part in vocational training, often ending up in jobs without formal qualifications [4]. Moreover, the majority of women born around 1930 received no vocational training [21]. Beginning with the post-war period, the average age of marriage decreased until about 1970 and afterwards increased [11], and the average age of women when bearing their first child increased in younger birth cohorts [16]. There was a peak in the number of children born from women who were born in 1933 with a decreasing trend across later birth cohorts (i.e., women born before 1966) [10]. Due to the end of WWII, many people immigrated to Germany as they had to flee from other, mainly Eastern European countries [28].

For comparison of age groups, one means for making sure enough individuals of a specific age are available for analysis in survey samples is to oversample rare individuals (e.g., older men); however, the small population number of individuals in oldest age effectively limits the degree of disproportionality that can be achieved in the actual sample, especially when the total sample size is large.

Because sample size and selectivity precludes a fuller picture of the heterogeneity of conditions that exists in this age group, current studies offer only limited potential to discuss normative aspects of QoL in the oldest old. In comparison to other ageing studies in Germany, NRW80+ is unique in that it includes individuals in care facilities and uses proxy interviews to represent those unable to answer questions themselves (e.g., due to cognitive impairment).

## The NRW80+ sample

NRW is the most populous state in Germany, counting 17.9 million inhabitants, imcluding 20% old individuals. Furthermore, NRW has a history of immigrants, making its population heterogeneous. The NRW80+ study was designed for robust inference about age group and gender differences and built upon the results of a comprehensive feasibility study [39]. A priori power analysis indicated that a sample size of *N* = 1548 would enable detection of small interaction effects (F = 0.1) between design factors (age group × gender) with high power (1-β = 0.95) at a conventional alpha level of 0.05. The population of the study included all people who had reached 80 years of age by 31 July 2017 and whose registered primary residence was in NRW. This includes individuals living in private and non-private settings (e.g., long-term care). The sampling followed a two-step procedure: First, a sample of 94 communities was drawn from the entirety of all communities in NRW. In a second step, the registration offices of the selected communities provided a simple random sample of inhabitants, amounting to 48,137 addresses from the target population. The group of potential study participants (gross sample) was defined to comprise *N* = 8040 individuals based on an a priori power analysis and an expected response rate of 20–25%. Individuals from older age groups (85–89 years, 90+ years) and men were systematically oversampled, i.e. represented more frequently within the gross sample than would be expected in a simple random sample (Table 1); however, equal sample size (*N* = 1340 or 16.7%) in each of the six design groups (i.e., age group × gender) was not feasible due to the low number of men aged 90 years or older (M90+) in the population.

**Table 1:** Sample composition with respect to age and gender distribution

Design group	Register sample (*N* = 48,137)	Gross survey sample (*N* = 8040)	Response rate^a^	Net survey sample (*N* = 1863)	Effective sample size
	%	%	%	N (%)	% (N)
*Male*					
80–84 years (M8084)	22.2	17.5	27.4	384 (20.6)	87.2 (335)
85–89 years (M8589)	10.7	14.7	25.9	299 (16.0)	84.9 (254)
90 years or older (M90+)	3.6	12.5	24.4	244 (13.1)	84.6 (206)
*Female*					
80–84 years (F8084)	32.6	20.0	21.6	344 (18.5)	88.3 (304)
85–89 years (F8589)	19.5	18.7	21.9	326 (17.5)	85.4 (278)
90 years or older (F90+)	11.4	16.7	20.2	266 (14.3)	85.2 (227)

Unweighted data

^a^percentage of realized interviews from all eligible cases

Computer-assisted personal interviews (CAPI) were conducted by experienced and trained interviewers of Kantar (previously TNS Infratest, Munich, Germany). A total of 1863 interviews were realized, assessing—besides QoL resources and outcomes—central events in the life course. Response rates were lower for older age groups and lower for women compared to men; however, a minimum of 244 observations could be realized for all design groups, allowing for robust subgroup analysis. Design weights were computed for all individuals selected into the gross sample to correct for selection of communities and oversampling of men and older age groups. Finally, calibration weightings were computed for participants in an iterative raking process based on the known demographic structure of the very old population with respect to age, gender, marital status, household size, institutionalization, and regional characteristics (for details *see* [9]). Even after applying weighting to correct for the disproportional sampling design and study nonresponse, effective sample size in all groups remained large. For example, the precision of population estimates in the strongly oversampled M90+ group in the NRW80+ sample is the same as the precision from a simple random sample of 206 individuals in this population group.

Respondents were on average 86.5 ± 4.5 years old (range 80–102 years) at the time of the interview. Table 2 shows that in the overall population of very old adults, 13.9% live in an institution. The number of very old individuals for which proxy interviews could be conducted was estimated at 8.8% in the population of the very old. Overall, only a minority of 33.2% of those 80 years or older show a formal need for care. Approximately half of the very old population showed medium levels of education, while high levels of formal education (i.e., bachelor’s degree and equivalent professional level or higher) were found for only one out of five persons in this age segment.

**Table 2:** Basic sample characteristics and estimated numbers in the NRW old age population

	NRW80+ wave 1 sample (*N* = 1863)	Population by 31 December 2016 (*N* = 1,077,296)
	*N %*	*N*
*Gender* (male)	676 *36.3*	390,702
Female	1187 *63.7*	686,594
*Age group* (80–84 years)	1012 *54.3*	585,050
85–89 years	573 *30.7*	331,145
90 years or older	278 *15.0*	161,102
*Living situation* (private)	1604 *86.1*	927,713
Living in institution	259 *13.9*	149,583
*Informant* (self-report)	1698 *91.2*	982,011
Proxy report	165 *8.8*	95,285
*Levels of care demand* (none)	1210 *66.8*	699,907
Level 1	58 *3.2*	33,304
Level 2	214 *11.8*	123,767
Level 3	193 *10.6*	111,500
Level 4	95 *5.3*	55,052
Level 5	43 *2.4*	24,849
*Education* (ISCED, low)	534 *30.0*	308,969
Medium (upper/post-secondary)	914 *51.3*	528,280
High (tertiary)	332 *18.6*	191,701

Weighted data

*ISCED* International Standard Classification of Education

**Fig. 1:**
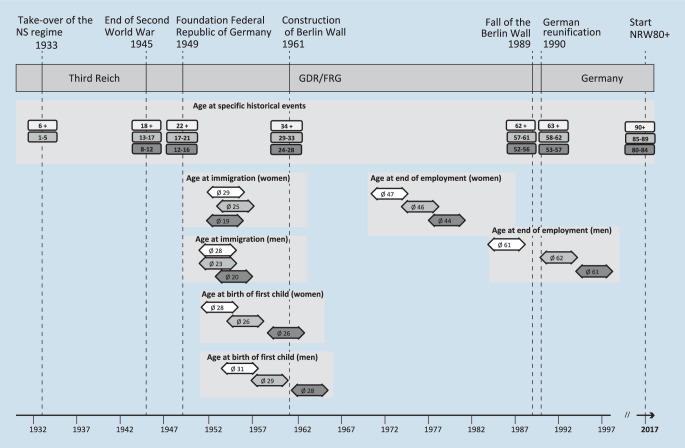
Timing of historical events in the life course of cohorts of the very old and differences with respect to age at key biographical events. *FRG* Federal Republic of Germany, *GDR* German Democratic Republic

Substantial age group differences were observed with respect to educational background (ISCED; [14]), employment history, socioeconomic status (International Socio-Economic Index of Occupational Status [ISEI]; [15]), marital status, institutionalization, birth of first child, and age at immigration (Table 3 and Fig. 1). The risk of institutionalization increased across age groups. Oldest individuals attained lower educational levels (i.e., up to lower secondary) in comparison to those in younger groups; however, most heterogeneity in educational level was attributable to gender. In the youngest age group, the share of women never having been employed was lower. Within the youngest age group and in women, divorce was more common. Furthermore, in the oldest age groups, more individuals were widowed. Of those having children, the oldest age groups (both men and women) were older when having their first child than the two youngest age groups (see Fig. 1). Of those who migrated to Germany, the youngest age groups were younger at arrival in Germany (19 and 20 years for women and men, respectively) with increasing age in those between 84 and 89 years and 90+ years. More than half of the NRW80+ participants who immigrated did so shortly after the Second World War. Whereas women were on average younger when ending employment than men, no substantial age group differences were observed in both men and women.

**Table 3:** Age group background characteristics and response behavior

% or M [95%CI]	80–84 years (*1933–1937)		85–89 years (*1928–1932)		90+ years (*–1927)		Test^a^
	Men	Women	Men	Women	Men	Women	
*Education* (ISCED, low)	9.1	33.4	9.9	44.5	9.6	41.8	Age: *p* = 0.041, Sex: *p* < 0.001, Age * Sex^c^: *p* = 0.172
Medium	56.1	55.9	53.0	44.2	61.8	50.8	
High	34.8	10.7	37.1	11.3	28.6	7.4	
*Employment* (past employment)	99.8	92.2	99.8	85.5	100	87.3	Age: *p* < 0.01, Sex: *p* < 0.001, Age * Sex: *p* < 0.001
Never employed	–	7.8	–	14.0	–	12.4	
Still employed	0.2	–	0.2	0.5	–	0.3	
*Socioeconomic status* (ISEI)	45.9 (43.6–48.2)	37.7 (35.5–39.9)	47.1 (43.9–50.2)	36.0 (33.5–38.4)	49.4 (46.0–52.9)	39.6 (37.0–42.1)	Age: *p* = 0.049, Sex: *p* < 0.001, Age * Sex: *p* = 0.466
*Marital status* (married)	71.9	32.8	59.3	12.2	46.7	5.4	Age: *p* < 0.001, Sex: *p* < 0.001, Age * Sex: *p* < 0.001
Married, but separated	1.7	1.3	0.7	0.2	0.4	–	
Divorced	3.9	6.0	3.1	3.6	0.7	3.0	
Widowed	18.9	55.4	35.9	77.8	48.8	85.7	
Single	3.7	4.5	1.0	6.2	3.5	6.0	
*Living situation* (private)	95.9	92.1	88.3	80.4	82.6	59.5	Age: *p* < 0.001, Sex: *p* < 0.001, Age * Sex: *p* = 0.265
Living in institution	4.1	7.9	11.7	19.6	17.4	40.5	
*Migration status* (immigrated)	25.4	22.8	21.1	22.8	22.8	26.5	Age: *p* = 0.527, Sex: *p* = 0.646, Age * Sex: *p* = 0.410
*Age at birth of first child*	28	26	29	26	31	28	Age: *p* < 0.001, Sex: *p* < 0.001, Age * Sex: *p* = 0.683
*Age at immigration*	20	19	23	25	28	29	Age: *p* < 0.001, Sex: *p* = 0.830, Age * Sex: *p* = 0.618
*Age at end of employment*	61	44	62	46	61	47	Age: *p* < 0.114, Sex: *p* < 0.001, Age * Sex: *p* = 0.455
*Response behavior*^b^							
Don’t know (%)	1.2 [0.9–1.5]	1.5 [1.2–1.9]	1.8 [1.3–2.2]	2.5 [2.0–3.0]	2.4 [1.9–2.9]	3.5 [2.8–4.2]	Age: *p* < 0.001, Sex: *p* < 0.001, Age * Sex: *p* = 0.172
Refuse to answer (%)	1.9 [1.3–2.4]	1.6 [1.0–2.1]	2.1 [1.3–2.8]	1.9 [1.4–2.4]	2.2 [1.5–2.8]	2.4 [1.6–3.1]	Age: *p* = 0.071, Sex: *p* = 0.631, Age * Sex: *p* = 0.708

Weighted data

^a^Tests for main and interaction effects used Taylor linearization to account for the multistage sampling and linear, logistic, or generalized logistic modelling for metric, ordinal, or nominal dependent variables respectively

^b^Percentage of refusal to answer or “don’t know” responses of all questions asked at the level of the individual. Hence, differences in the number of questions asked at the level of the individual due to filtering are accounted for in the average score given in the table

^c^Interaction between age and sex

Item nonresponse measured at the level of the individual was generally low in this study. On average, less than 4% of all information asked from a respondent was lacking due to refusal to answer or “don’t know” responses. Nevertheless, while the share of person-level refusals did not increase across age groups, “don’t know” answers did. Part of this effect was due to the increasing share of interviews with proxy informants in older age groups; however, additional analysis showed that age had an independent (albeit small) effect on item nonresponse over and above the effect of proxy informant and cognitive status (standardized beta = 0.14, 0.35 and 0.34, respectively). Hence, item nonresponse in this study of the very old was rare and multifactorial. Besides, the prevalence of cognitive impairment estimated based on the NRW80+ sample was comparable to prior epidemiological findings [13]. Two out of three respondents showed age-adequate cognitive functioning according to norm data and a similar proportion of individuals were screened or rated as mild cognitive impaired (MCI) or early dementia.

## A theoretical framework of QoL in very old age

Even though a plethora of QoL studies exist on the individual, on a group or country level, and in many specific subpopulations [26], the QoL of very old individuals has rarely been examined and there are few QoL models focusing particularly on very old individuals [18]; however, existing studies [6, 7] suggest that in older people—compared to younger age groups—QoL is determined by different aspects. For example, meaningful, eudaimonic aspects seem to be important in older adults [12]. A detailed investigation of different determinants (e.g., personal, environmental, or their interaction) of very old individuals may help to understand unexpected results, such as the well-being paradox in old age. For example, Schilling [34] found that the well-being paradox in old age (i.e., seemingly stable levels of well-being with decreasing levels of resources [36]) results from a change in health resources as well as differences between cohorts with regard to life satisfaction. In addition, it may be important to identify cohort-specific determinants of QoL in very old age, as early socialization or differences in the timing of major life events (e.g., education, childbearing, retirement) have been found to impact QoL at older ages (e.g. [23, 30]).

**Fig. 2:**
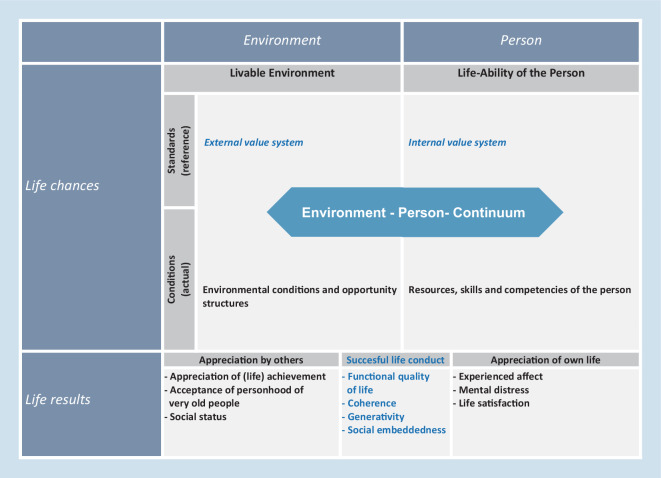
Challenges and Potentials Model of Quality of Life in Very Old Age (CHAPO, 40)

With respect to a broad understanding of QoL in very old age, Wagner et al. [40] proposed a framework to integrate major streams of research on subjective aspects of psychological well-being (e.g., life satisfaction) as well as the scientific investigation of the (societal) basis of economic welfare (e.g., education or income). The “Challenges and Potentials Model of Quality of Life in Very Old Age” (CHAPO; [40], see Fig. 2) was based on Veenhoven’s model [38] adding values residing within an individual and/or within the environment (e.g. societal values) as QoL resources. Individual and perceived societal values are assessed in the form of interview questions, whereas a separate qualitative study evaluated the societal perspective based on stakeholder interviews. The CHAPO was developed as a conceptual framework to operationalize resources and outcomes that are central to the interdisciplinary discussion of QoL in very old age. Given the heterogeneity resulting from vastly distinct life courses of today’s very old population, individual values may be idiosyncratic or not congruent with the values of others, younger generations, or today’s society, creating a tension between societal groups with respect to the definition of QoL and successful aging.

Furthermore, CHAPO conceptually adds to existing frameworks of QoL in that it explicitly acknowledges the fact that successful life conduct—as a systemic QoL outcome—depends both on resources and values of the older individual as well as on roles and appreciation of late life by society. It allows for descriptive, evaluative, and normative perspectives on QoL in very old age (for a detailed description *see* [29]). Whereas other QoL models postulate specific mechanisms that promote or prevent QOL, CHAPO—at first sight—distinguishes QoL resources as potential predictors for QoL outcomes; however, it should primarily be understood as a generic measurement model, serving as a basis to categorize indicators as life chances or life results and distinguishing personal from environmental indicators. Nevertheless, the operationalization of NRW80+ built on previous empirical evidence to include indicators particularly relevant for this age segment. With regard to life chances, indicators in NRW80+ include individual values (*see* [32] in this issue) or social relations (*see* [35] in this issue) for the person and environment level, respectively. Life results included indicators such as life satisfaction (*see* [8] in this issue). CHAPO adds to this the notion of successful life conduct as a systemic concept integrating the idea of person-environment-fit and mechanisms to retain identity, autonomy, and participation in light of compromised physical and mental capacity that characterize fourth age [42–44]. Here, fit refers to a specific positive constellation of resources and demands that foster functionality, independence, or personal growth. Successful aging [37] is defined by an autonomous, generative, active, or productive behavior by using respective educational, social, infrastructural, technical, or economic resources.

Indicators and determinants of QoL are assumed to be different even across age groups within very old age for a number of reasons. First, very old age today is predominantly female and gender differences for QoL predictors and indicators have to be considered [31]. Second, individuals in their beginning 80s may not (yet) experience a drastic decrease in individual resources (e.g., health, social network) and consequently depend less on environmental resources for QoL; however, the relative contribution of environmental resources for autonomy and QoL may be greater in the oldest old.

## Discussion

The NRW80+ study allows making robust statements about age group differences within the population segment of very old adults and strengthens the state of research on quality of life of the oldest old in Germany. The sampling strategy was successful in guaranteeing a high level of precision of population estimates, particularly in the rare and hard to reach group of men aged 90 years or older and sufficient power to test the small to moderate effects expected in social-behavioral aging research.

Age groups within very old age differed substantially with respect to health status, education, past employment, socioeconomic and marital status, resulting in very diverse conditions for and circumstances of realizing successful life conduct.

Results showed differences in the timing of major life events across different age groups within very-old age. The particular age at which significant life transitions (e.g., childbearing) were experienced may influence subsequent biographies and QoL in very old age. For example, immigration at different ages may have consequences for the integration into a new community and therefore may impact QoL; however, several limitations of the current data are noteworthy. Firstly, operationalization of QoL focused on current status and offered only a limited window to study biographical antecedents. Secondly, with cross-sectional data, disentangling age or cohort effects was severely limited. Finally, individuals who survived up to a very old and oldest age can be expected to represent a specific subgroup of the respective birth cohorts. Finally, the face of very old age is changing quickly. The share of very old men, for example, is expected to increase substantially across the next decades.

## Conclusion

The NRW80+ study offers a unique possibility to investigate QoL in a representative sample of very old adults from the most populous state in Germany. Whereas the share of older people in the German population increases, representative studies about QoL of this age group remain rare.

The NRW80+ study meets a number of conceptual and methodological challenges of conducting a survey on QoL in the very old population. The CHAPO model considers eudaimonic concepts of QoL as well as concepts integrating personal and environmental aspects especially relevant in old age. A specific strength of this study is the possibility of distinguishing age groups of privately and nonprivately dwelling individuals within very old age, whose differences in socialization, education, and life experiences should exert profound impact on late life QoL outcomes. Hence, the NRW80+ study identifies needs and determinants upon which policy recommendations can be made to create conditions in which individuals may realize and retain successful life conduct throughout late life.

## Practical implications

- A scientific use file of the NRW80+ wave 1 data is available at the GESIS data repository (10.4232/1.13527).
- A second wave of the NRW80+ study including more than 900 interviews of wave 1 participants and more than 900 additional initial interviews in a new random sample of individuals 80+ was completed in March 2021. Data will be made available at the GESIS data repository.
- The NRW80+ study protocol was adopted for the Bundesministerium für Familie, Senioren, Frauen und Jugend (BMFSFJ)-funded national study on the oldest old D80+ “Hohes Alter in Deutschland” conducted in cooperation with the German Centre of Gerontology (DZA) in Berlin.

## Declarations

This study was carried out in accordance with the ethical standards of the ethics committee of the Medical Faculty of the University of Cologne and with the Helsinki Declaration of 1975 (in its most recently amended version). Informed consent was obtained from all participants included in the study.
